# Providing emergency care and assessing a patient triage system in a referral hospital in Somaliland: a cross-sectional study

**DOI:** 10.1186/s12913-014-0531-3

**Published:** 2014-11-06

**Authors:** Temmy Sunyoto, Rafael Van den Bergh, Pola Valles, Reinaldo Gutierrez, Latifa Ayada, Rony Zachariah, Abdi Yassin, Sven Gudmund Hinderaker, Anthony D Harries

**Affiliations:** Médecins Sans Frontières – Operational Centre Brussels, Somaliland Mission, Hargeisa, Somaliland; Médecins Sans Frontières – Operational Centre Brussels, Operational Research Unit (LuxOR), Luxembourg, Grand Duchy of Luxembourg; Médecins Sans Frontières – Operational Centre Brussels, Brussels, Belgium; Ministry of Health, Togdheer Region, Somaliland; Centre for International Health, University of Bergen, Bergen, Norway; International Union Against Tuberculosis and Lung Disease (The Union), Winchester, UK; Médecins Sans Frontières, Operational Centre Barcelona, Delhi, India

**Keywords:** Emergency care, Resource-poor, Triage, SATS, Somaliland

## Abstract

**Background:**

In resource-poor settings, where health systems are frequently stretched to their capacity, access to emergency care is often limited. Triage systems have been proposed as a tool to ensure efficiency and optimal use of emergency resources in such contexts. However, evidence on the practice of emergency care and the implementation of triage systems in such settings, is scarce. This study aimed to assess emergency care provision in the Burao district hospital in Somaliland, including the application of the South African Triage Scale (SATS) tool.

**Methods:**

A cross-sectional descriptive study was undertaken. Routine programme data of all patients presenting at the Emergency Department (ED) of Burao Hospital during its first year of service (January to December 2012) were analysed. The American College of Surgeons Committee on Trauma (ACSCOT) indicators were used as SATS targets for high priority emergency cases (“high acuity” proportion), overtriage and undertriage (with thresholds of >25%, <50% and <10%, respectively).

**Results:**

In 2012, among 7212 patients presented to the ED, 41% were female, and 18% were aged less than five. Only 21% of these patients sought care at the ED within 24 hours of developing symptoms. The high acuity proportion was 22.3%, while the overtriage (40%) and undertriage (9%) rates were below the pre-set thresholds. The overall mortality rate was 1.3% and the abandon rate 2.0%. The outcomes of patients corresponds well with the color code assigned using SATS.

**Conclusion:**

This is the first study assessing the implementation of SATS in a post-conflict and resource-limited African setting showing that most indicators met the expected standards. In particular, specific attention is needed to improve the relatively low rate of true emergency cases, delays in patient presentation and in timely provision of care within the ED. This study also highlights the need for development of emergency care thresholds that are more adapted to resource-poor contexts. These issues are discussed.

**Electronic supplementary material:**

The online version of this article (doi:10.1186/s12913-014-0531-3) contains supplementary material, which is available to authorized users.

## Background

The Emergency Department (ED) is a crucial entry point to health services for patients requiring urgent and immediate care [1]. Delays in providing rapid and effective care to such patients may result in death or permanent disability [2,3]. Where there are challenges at the level of poor infrastructure, limited human resources, or overburdened health systems, such as in many resource-constrained and/or post-conflict settings, access to emergency care is limited [4,5].

One approach to reducing the strain on overburdened emergency services is the use of a proper emergency triage tool, which is intended to ensure that patients receive the most appropriate level and quality of care relative to their clinical status and need, thus ensuring optimal use of clinicians’ time and resources. The South African Triage Scale (SATS) is such a tool. This was developed for use used by non-specialist (nursing) staff to identify patients at higher risk of death, and thus to enhance ED efficiency [6]. In the centres where it has been evaluated – such as urban and rural centres in South Africa, where it was developed [6,7], and elsewhere [8] – it has been associated with positive outcomes such as reductions in waiting time, length of stay, and mortality [9], and, at the same time, improvement of the patient flow.

In general, studies on ED utilization and performance have mainly focused on specific medical areas such as paediatrics [10] or have taken place in relatively well-resourced contexts [11], and few studies have analysed the performance of emergency care services in developing country settings. Specifically, there is a dearth of evidence on the feasibility and utility of the SATS tool in resource-limited settings, where emergency care services are overburdened and no pre-existing systematic triage systems are in use.

Somaliland, a self-declared independent region of Somalia, is an example of a resource-constrained context where access to emergency care is limited. Following decades of civil war, the health infrastructure is dilapidated, trained health care workers are scarce, and the health indicators are poor [12]. In the public hospital of Burao town, Togdheer region of Somaliland, with the support of the medical humanitarian non-governmental organization (NGO) Médecins Sans Frontières (MSF), an ED was started in early 2012. Prior to this, there was no provision of emergency care except through the maternity department for emergency obstetric cases.

This study aimed to describe the feasibility of managing an ED, including implementation of the SATS, in a district referral hospital in Somaliland during its first year of service. Specific objectives were to assess emergency service utilization, the capacity to meet the pre-set indicators of the SATS tool, and outcomes of patients seen in the ED in such a context.

## Methods

### Design

This was a descriptive, cross-sectional study with follow-up data on discharge outcomes, using data from routinely collected records.

### Setting and population

Somaliland, a breakaway region of Somalia situated in the north-west of the Horn of Africa, has a population of approximately two million, with 43% of the people living on less than 2 United States Dollars (US$) a day [12]. Somaliland, not officially recognized as an independent country by the international community, is still mired in political conflicts and suffers from a dysfunctional health system.

Burao General Hospital is a 140-bed hospital located in the Togdheer region of Somaliland, serving approximately 400,000 people, the majority of whom are nomadic pastoralists [13]. There is no referral network or functional ambulance system in place. Burao General Hospital serves as the only secondary level public health care facility in the region, offering comprehensive services including outpatient consultation, inpatient hospitalization (maternity, paediatric wards, medical and surgical ward), operating theatres, blood transfusion and laboratory services. No radiography or intensive care unit is available. Since March 2011, MSF has collaborated with the Ministry of Health to improve the quality of hospital care and to increase access to care by providing services free of charge.

The ED of Burao General Hospital underwent rehabilitation and became operational in January 2012– it was provided with only basic equipment. From the outset, the ED was staffed by three nurses in the morning and two in the afternoon and night, one national doctor, and one expatriate doctor working daily from 8 am to 5 pm, except on Fridays. A training programme was conducted for the ED staff, covering the basics of emergency care through formal sessions and on-the-job training. The triage is typically done by nursing staff. Patients are self-referred to the ED, and on assessment are either admitted or referred to other services.

The SATS was implemented since the opening of the ED, with training on SATS conducted for the ED nurses and doctors from the outset. The SATS uses a physiologically based scoring system, the Triage Early Warning Score (TEWS), and a list of discriminators designed to triage patients into one of five colour-coded priority groups for medical attention. An adapted version of the tool is available for adults, children and infants. Details of the triage system are shown in Additional file 1.

### Study population

All patients (adults and children) presenting to the ED of Burao General Hospital and recorded in the electronic ED register from January to December 2012 were included in this study.

### Outcome variables

The main variables collected for the purposes of this study were the daily numbers of patients attending the ED, and the individual SATS scores (colours); surveillance diagnoses; the timings of arrival/triage, clinical consultation and discharge; and the patient ED outcomes.

The *surveillance diagnosis* was recorded according to programme definitions, which included trauma (accidental), trauma (violent), acute abdominal conditions, obstetrics/gynaecological conditions, asthma/chronic obstructive pulmonary disorder (COPD) exacerbation, cardiovascular emergencies, respiratory tract infections, hyperglycaemic crisis, and others. The *outcome* was defined as: “Discharged”: a patient who left the ED straight to home; “Admitted”: a patient who left the ED and went to another department of the hospital, such as an inpatient ward or operating theatre; “Defaulted” or ‘Abandoned from hospital setting’: a patient who left the ED against/without medical advice (set threshold <5%); or “Died”: a patient who died inside the ED (set threshold <1%). Time of arrival in the ED/triage and time of clinical consultation were recorded by the ED nurse: these were compared to the target times described in Additional file 1 to classify patients as seen on time or not.

Indicators that were used to assess the SATS performance were 1) *high acuity proportion*, or proportion of cases with a high level of urgency, defined as “all red and orange cases/all triaged cases”, with a target of being ≥25%; 2) *overtriage*, or proportion of cases incorrectly triaged as very urgent, defined as “discharged red and orange cases/all red and orange cases” with a threshold of <50%; and 3) *undertriage*, or proportion of cases incorrectly triaged as less urgent, defined as “admitted, referred or died green cases/all green cases” with a threshold of <10%. These thresholds have been set in line with international guidelines developed by the American College of Surgeons Committee on Trauma (ACSCOT) [14,15].

All data were collected using a patient electronic register and individual patient charts situated in the hospital.

### Data collection and analysis

A retrospective audit of the electronic data register based on individualized ED patient charts from 1 January 2012 to 31 December 2012 was conducted. The patient chart contains all the variables including time of arrival and consultation. Data were collected into a dedicated Excel database, and were analysed using EpiData v.2.2.1.171 software (EpiData Association, Odense, Denmark). Descriptive analysis was performed and differences of proportions were assessed using chi-square test where appropriate.

### Ethics

This study met the criteria approved by the Médecins Sans Frontières’ Ethics Review Board (Geneva, Switzerland) for analysis of routinely-collected program data, and was also approved by the Ethics Advisory Group of the International Union Against Tuberculosis and Lung Disease, Paris, France. Approval was also obtained from the local Ministry of Health and hospital authorities, as no formal ethics body exists in Somaliland.

## Results

**Figure 1:**
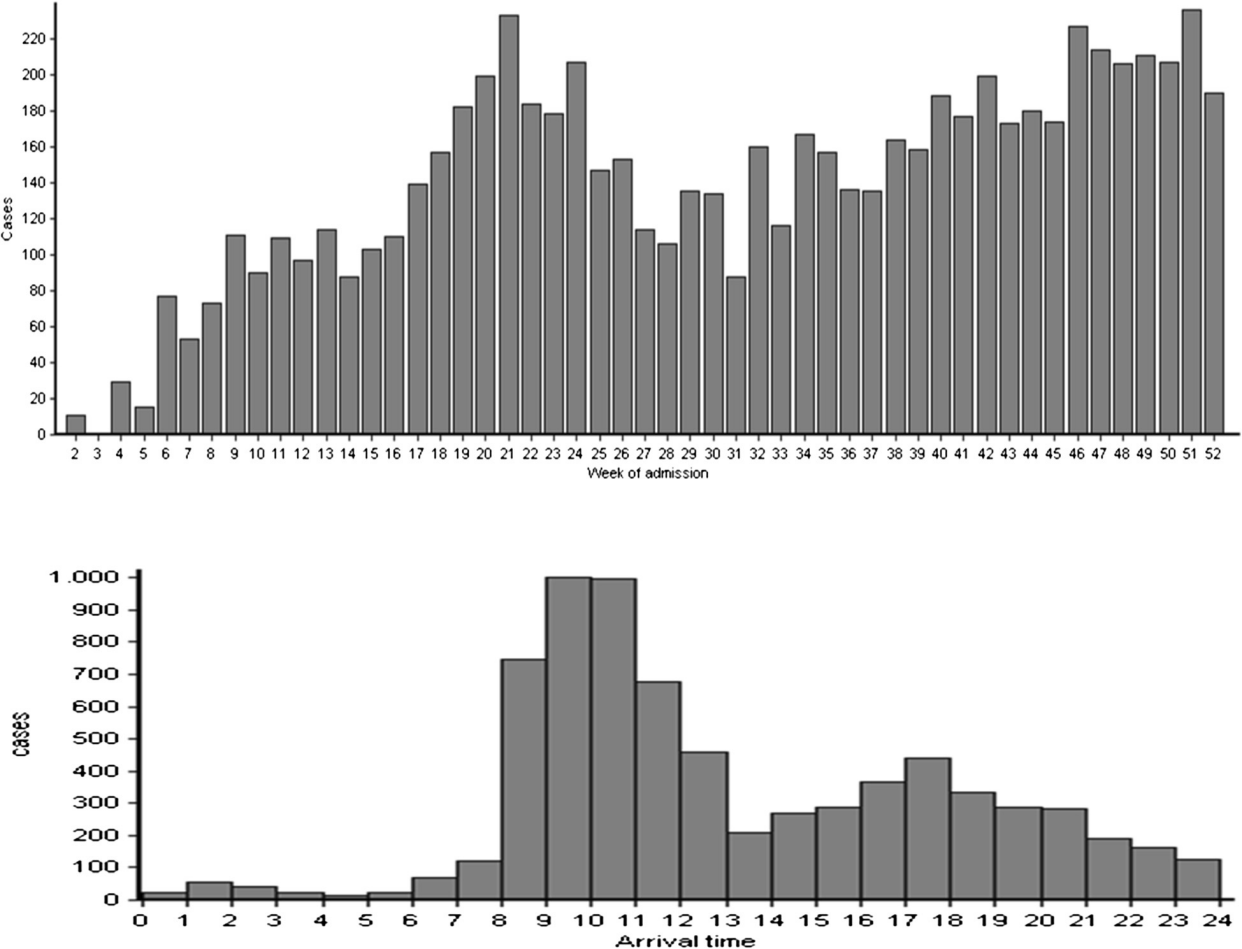
**Weekly admission trends in the Burao General Hospital ED, January-December 2012 (top) and Admission trend per 24 hours in the Burao General Hospital ED, January-December 2012 (bottom).**

In 2012, there were 7212 consultations conducted in the ED of Burao General Hospital. The trend is depicted in Figure 1. There was a steady increase in the first months during the start-up phase, and a decline around July-August, coinciding with the Ramadan or fasting period which is widey observed in Somaliland. Peak days with more than 40 cases per day were occasionally noted (n = 9). Patients tended to arrive in rush hours, peaking between 9 am and 11 am, and again to a lesser extent between 4 pm and 6 pm.

**Figure 2:**
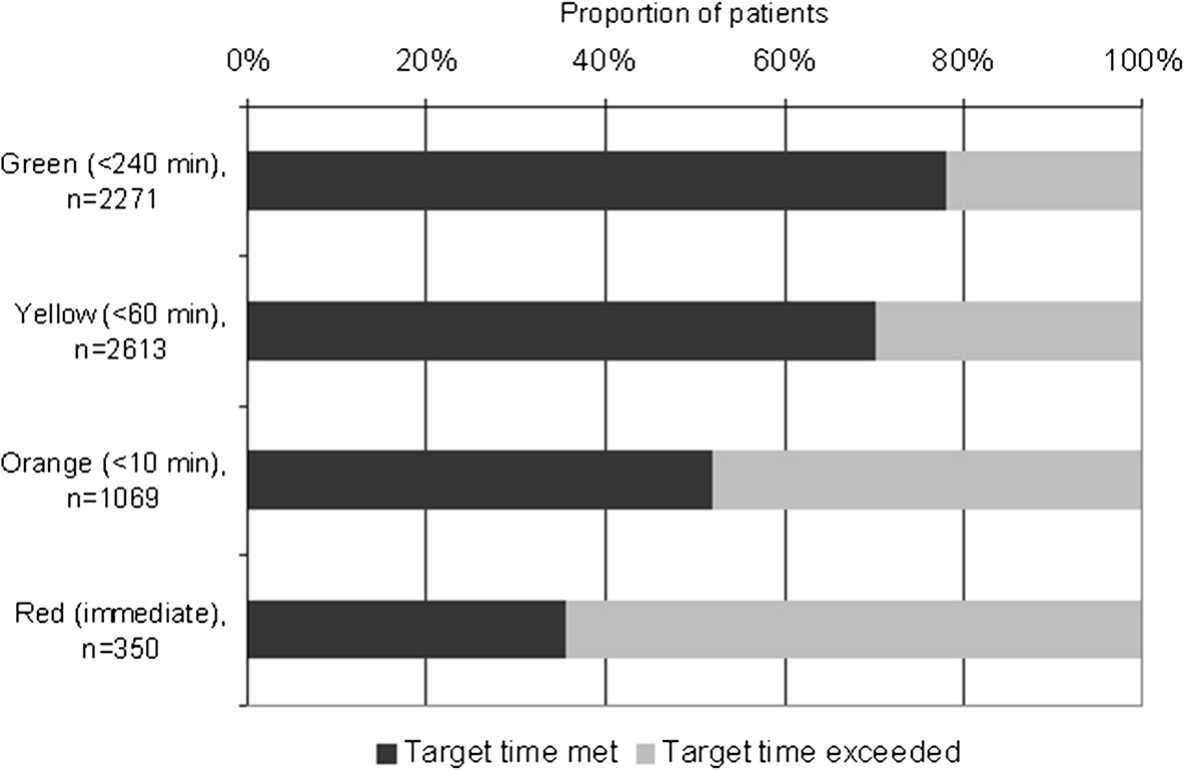
**Proportion of patients who were treated within the target time (as determined by their SATS score) in the Burao General Hospital ED, January-December 2012.**

Out of the 7212 patients attending the ED, 2962 (41%) were female, and 1305 (18%) were aged less than five. Admission and management characteristics of all patients presenting at the ED are provided in Table 1: red and orange cases represented 6% and 17% of all patients respectively, indicating a “high acuity” proportion of 22.3% (95% CI 21.3% - 23.3%). Delays in presentation at the ED after development of symptoms were observed: out of 5017 patients for whom data were available on timing of onset of symptoms, only 21% of the patients attended the ED within 24 hours after developing symptoms. Delays in receiving care after presentation at the ED were also observed: proportions of patients treated within the target time to treat (cf. Additional file 1) are indicated in Figure 2. The “immediate” target time to treat for red cases was exceeded in more than 60% of the cases, and only half of the orange cases received treatment within the target time of 10 minutes. Patients for whom the target time to treat was not met were at increased risk of death, with a Relative Risk of 2.2 (95% CI 1.4-3.4,) when compared with all patients treated within time, although the difference was not significant when stratified for SATS score.

**Table 1:** **Characteristics of patients accessing the Burao General Hospital ED, January-December 2012 (n = 7212)**

**Characteristics**	**n (%)**
**SATS score**	
Red	399 (6)
Orange	1208 (17)
Yellow	2968 (41)
Green	2590 (36)
Blue	15 (0.2)
Not recorded	32 (0.4)
**Morbidities (top 10)**	
No surveillance diagnosis	2217 (31)
Trauma (accidental)	1508 (21)
Watery diarrhoea	743 (10)
Lower respiratory tract infection	654 (9)
Trauma (violent)	551 (8)
Measles	277 (4)
Upper respiratory tract infection	212 (3)
Acute abdominal conditions	205 (3)
Cardiovascular diseases	154 (2)
Asthma/COPD* exacerbation	110 (2)
**Time interval symptoms-presentation (n = 5017)**	
<12:00 h	906 (18)
12:00–23:59 h	124 (2)
1-7 days	2434 (49)
8 days – 1 month	990 (20)
>1 month	563 (11)

*COPD: chronic obstructive pulmonary disorder.

**Figure 3:**
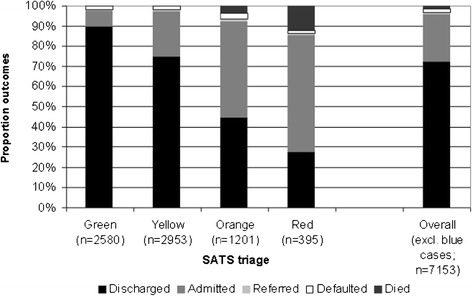
**Proportional outcomes in relation to the SATS score of patients in the Burao General Hospital ED, January-December 2012.**

Outcomes of patients in relation to their SATS score (excluding cases who were dead on arrival – blue SATS cases) are shown in Figure 3. The overall ED mortality rate was 1.3% (95 cases, 95% CI 1.0% - 1.6%) and “default” or “abandon” rate was 2.0% (142 cases, 95% CI 1.7% - 2.3%). Hospital admission and mortality rates were correlated with the degree of urgency, as expected; overtriage (40%) and undertriage (9%) remained under the pre-set thresholds (50% and 10% respectively). Leading causes of death were cardiovascular diseases (16 cases, 17%), lower respiratory tract infections (13 cases, 14%), and accidental trauma (11 cases, 12%). Additionally, 27 deaths (29%) with a surveillance diagnosis of “Other” were observed.

## Discussion

This study demonstrates the feasibility of providing emergency care, including implementation of the new SATS triage system in Burao General Hospital, Somaliland.

The utilization of the ED increased steadily during its first year of service, but late presentation at the ED was observed, with only 21% of patients presenting within 24 hours after developing symptoms. Utilization patterns of an ED are influenced by many factors, including the structure, the finance mechanism, geographical distance, perceived quality, access and availability of alternatives [16,17]. However, the sociodemographic aspects related to the utilization pattern seen in Burao is not well understood. The high proportion of late presenters to the ED suggests considerable barriers to care and lack of awareness amongst patients and the community on the need of early presentation. Absence of emergency transport has been identified as a major barrier in accessing emergency care [1,18] and could certainly play a role in our setting, but the evidence collected over the course of this study did not allow us to address this issue.

The observed late presentation may also be associated with the relatively high rates of non-emergency cases (demonstrated by the high acuity rate of 22.3%, falling short of the set target): it is possible that the ED was perceived as complementary to the conventional outpatient services, and was frequented by patients with more general and long-standing complaints. These patients arrive at the hospital outside of the outpatient department opening hours which is only between 9 am to 12 pm. The provision of ED services free-of-charge, as opposed to the outpatient services provided at cost, may also have led to a preference for the ED. In general, differing perceptions between ED patients and health staff on the severity and urgency of medical problems have been shown to be associated with ED overcrowding by non-emergency cases [19,20]. Many patients accessing hospital level emergency centres have been shown to bypass the primary health care system [21,22], and as the primary health centres in the region do not provide emergency care, this is likely to be the case for the ED in Burao hospital. Further qualitative analysis on the demand of emergency care could inform better decisions on health care resources allocation.

The surveillance diagnoses of all presenting cases show that trauma (both accidental and violent) contributes to 29% of total cases, indicating that injuries remain an important cause of seeking emergency care [2,3]. Both unintentional and intentional injuries are acute events by definition, therefore nearly all require emergency care, and the disease burden caused by injuries is amenable to emergency care [5].

Patient outcomes were unsurprisingly associated with their triage categories, with higher discharge rates among the least urgent conditions (coded green and yellow), and admission and death occurring more frequently for red and orange cases. The overall “default” or “abandon” rate (2%, 95% CI 1.6-2.3%) was well under the threshold of 5%; while the mortality rate (1.3%) slightly exceeded the threshold of 1%. Improved triage and emergency care have been shown to reduce inpatient mortality in Malawi and South Africa [7,10,], while also drastically reducing patients’ waiting times [6]. Poor triage on the other hand can jeopardize the life of patients arriving in the hospital [9,23]. An improved understanding of the delays in providing clinical consultation, in particular among high acuity cases, is therefore required, in order to address these delays and thus potentially reduce mortality.

Even during the first year of implementation, the targets of the SATS tool for overtriage and undertriage were met, indicating that the ED staff was able to use this system accurately and reflecting positively on the training programme provided. These results are similar to those reported for the evaluation of SATS in both urban and rural settings in South Africa [6,24], and for the implementation of the SATS in an ED in Pakistan [8]. As the SATS thresholds, taken directly from the ACSCOT guidelines, appeared readily achievable, even during the first year of ED operation, it is perhaps worthwhile reflecting whether these standards need adjustment to the context. In Somaliland and other less developed settings, the triage system is mostly used by nursing staff as there is a scarcity of emergency physicians [24,25], and setting the thresholds higher from the standards set in more developeed ED would actually make sense. Different performance indicators that are easily adjusted to the local context would further improve outcomes and utilization of emergency care services [26].

This study is the first to report on the functioning of an ED in a district hospital in a post-conflict, resource-constrained context such as Somaliland. With the paucity of empirical data on emergency care, especially on appropriate triage systems that ensure efficient and timely utilization of existing resources, the results of this study provide lessons learned that may be applicable to similar settings. The experience of establishing an ED in Burao Hospital shows the importance of several key components: a physical infrastructure with a good patient flow, training of health care providers with continuous supervision, and the choice of an effective use of a triage system.

The limitations of the study include its cross-sectional nature and the use of routinely collected data. Patient outcome was used as an indicator of the urgency of the patient condition at assessment, but outcomes remain a proxy indicator at best: no data was available on the true severity of the patient condition. However, outcomes do represent a robust proxy measure of the urgency of the patient condition in the ED. This has been established through ASCOT indicators, which looks specifically into acuity (if red and orange cases make up more than 25% of all cases triaged), and more practically towards undertriage or overtriage in the emergency department. No assessment of the impact of the programme could be performed as no data from before the ED was operational were available and quality improvements such as a reduction in mortality rates or in waiting times for the patients could not be demonstrated. The study also only assessed emergency care provision at the health facility, and did not include the pre-hospital care as part of the emergency care continuum.

There are still many gaps in global knowledge on providing emergency care in developing countries. Qualitative research is needed, for example to understand the reasons for late presentation to the ED or its use by non-urgent cases. Different health promotion or communication interventions, aiming to increase early presentation and presentation of true emergency cases, should also be undertaken, followed by proper quantification of their impact.

## Conclusion

The ED in Burao General Hospital was used by the population, suggesting that the provision of a basic but effective level of emergency care was feasible in this resource-limited setting. The ED responded to perceived and actual community needs and probably improved the health of the population. In Somaliland, where no national triage system exists and where resources are limited, the experiences described in this study provide baseline data and insights into the utilization pattern and patient outcomes of an ED in its first year: there is room for improvement and this could be measured in the future by assessing outcomes against interventions. Overall, our results indicate that the SATS was a user-friendly tool with satisfactory results when implemented in the ED in Burao, Somaliland.

## Additional file

Additional file 1:
**South African Triage Scale System.**
