# Supplementary material for: Providing emergency care and assessing a patient triage system in a referral hospital in Somaliland: a cross-sectional study
Source: BMC Health Serv Res. 2014 Nov 6;14:531. doi: 10.1186/s12913-014-0531-3 (PMC4229595; doi:10.1186/s12913-014-0531-3)
Supplement: Additional file 1: — South African Triage Scale System. [file 12913_2014_531_MOESM1_ESM.doc]

**Additional File**

**Box 1**

**South African Triage Scale System**

| Triage is a system to ‘’sort’’ out patients in order of urgency of treatment - the end result is that the patient with the greatest need is helped first.  The **South African Triage Score** is a triage tool that consists of calculating a Triage Early Warning System (TEWS – previously named the Cape Triage Score/CTS) and a list of conditions called a ‘’discriminator list’’, which are then combined to designate a color code which determine urgency of care. The TEWS consists of different variables: mobility, Respiratory rate, Heart rate, Systolic blood pressure, Temperature, consciousness level using AVPU, Trauma. The series of discriminators consist of Mechanism of injury, Presentation, and Pain. Based on an easy-to-read table, the triage officer assigns the final colour code.  There are 3 versions of SATS: adult (>12 yrs, or >150 cm tall), children (3-12 yrs old, 95-150 cm) and infant (0-3 yrs, <95 cm), each with its specific discriminator list but the final code categorization is the same:   - Red: scored 7 or more, target time to treat: immediate - Yellow: scored 5-6, target time to treat: less than 10 minutes - Orange: scored 3-4, target time to treat: less than 60 minutes - Green: scored 0-2, target time to treat: less than 240 minutes - Blue: dead on arrival |
| --- |
